# Study of the strongest dust storm occurred in Uzbekistan in November 2021

**DOI:** 10.1038/s41598-023-42256-1

**Published:** 2023-11-16

**Authors:** Bakhriddin E. Nishonov, Bakhtiyar M. Kholmatjanov, Lev D. Labzovskii, Natella Rakhmatova, Lyudmila Shardakova, Erkin I. Abdulakhatov, Darkhon U. Yarashev, Kristina N. Toderich, Temur Khujanazarov, Dmitry A. Belikov

**Affiliations:** 1 Hydrometeorological Research Institute, Center of Hydrometeorological Service of the Republic of Uzbekistan, 100052 Tashkent, Uzbekistan; 2 Faculty of Hydrometeorology, National University of Uzbekistan, 100174 Tashkent, Uzbekistan; 3 R&D Satellite and Observations Group, The Royal Netherlands Meteorological Institute (KNMI), De Bilt, The Netherlands; 4 International Platform for Dryland Research and Education (IPDRE), Tottori University, Tottori, 680-0001 Japan; 5 Disaster Prevention Research Institute, Kyoto University, Gokasho, Uji 611-0011 Japan; 6 Center for Environmental Remote Sensing, Chiba University, Chiba, 263-8522 Japan

**Keywords:** Atmospheric chemistry, Environmental monitoring, Atmospheric science, Climate-change impacts

## Abstract

We studied and reconstructed a severe Central Asian dust storm of November 4, 2021, through high-resolution TROPOMI UVAI spaceborne observations, ground-based aerosol measurements, and Lagrangian particle modeling. The dust storm was caused by the front part of a cold polar anticyclone front from the Ural-Volga regions, which struck the central and eastern parts of Uzbekistan under favorable atmospheric conditions. Two plumes spread out, causing a thick haze to blanket the region. The most severe dust storm effects hit the capital of Uzbekistan (Tashkent) and the Fergana Valley, where the thick atmospheric dust layer dropped the visibility to 200 m. PM_10_ concentrations reached 18,000 µg/m^3^ (260-fold exceedance of the local long-term average). The PM_2.5_ concentrations remained above 300 µg/m^3^ for nearly ten days, indicating an extremely long-lasting event. The dust storm was caused by an extremely strong summer heatwave of 2021 in Kazakhstan with unprecedentedly high temperatures reaching 46.5 °C. The long-lasting drought dried up the soil down to 50 cm depth, triggering the soil cover denudation due to drying out vegetation and losing its moisture. This event was the worst since 1871 and considering the increasing aridity of Central Asia, the onset of potentially recurring severe dust storms is alarming.

## Introduction

Dust and sandstorms have been traditionally viewed as localized phenomena, occurring mainly in arid and semi-arid regions and exhibiting mostly local-scale effects. However, under global warming, some dust storms (DS) have attained massive scales since the 1990s, thus prompting growing global concerns^1,2^. DSs are officially defined by the World Meteorological Organization (WMO) as the result of surface winds that raise large amounts of dust into the air and reduce visibility to less than 1000 m^3–5^. DS can lift thousands of tons of dust into the atmospheric boundary layer, which not only affects natural ecosystems but also disturbs the daily life of people and inflict economic damage by disrupting logistic/transportation chains, triggering aircraft/motor accidents, and even plaguing crop productivity^6–10^. Moreover, dust particles can weaken soil fertility, reduce insulation at the surface (which, in turn, reduces the output from solar devices) damage telecommunications, and mechanical systems, and cause or exacerbate respiratory diseases among people^7,9–17^.

Despite their global effects, massive DSs occur and develop only in a few regions worldwide with arid or semi-arid climates such as Sahara, Middle East, and Central Asia^18,19^. These regions experience low precipitation (mean precipitation < 200–300 mm/year), strong winds, scarcity of vegetation cover, long dry summers, and frequent repetition of soil and atmospheric droughts^1,20,21^. While severe DSs of the Middle East and Sahara have been thoroughly studied in previous reports, the studies on DSs of Central Asia and, let alone, their drivers, are very scanty. Central Asia is a vast region, primarily composed of arid ecosystems containing abundant mineral and salt aerosols, which, when lifted, can facilitate the development of severe DSs^13,19,22–24^. On top of that, since the 1960s, the region has experienced major land use changes, which exacerbated its aridity. For instance, in Kazakhstan, land degradation was initiated in the 1950–1960s due to the development of so-called “Pristine Lands”—the activities aimed to increase the number of animals and to alter local grazing practices^25^. At the same time, Turkmenistan, and Uzbekistan both suffered from land degradation due to the irrigated agriculture expansion programs of Soviet times. These programs have transformed the vast areas of natural desert pastures into cotton fields^26,27^. What is worse, the irrigation expansion program triggered a staggering reduction in water inflow to the Aral Sea^28,29^. As a result, the Aral Sea area has dramatically shrunk by 60,156.50 km^2^ (about 87.85%), thereby leading to a colossal total loss of water volume of ~1000.51 km^3^ in 1960–2018 period^30^. Nowadays, the Aralkum Desert appeared at the exposed bottom of the sea is the “distributor” of salts and chemicals, thereby adding a considerable amount of aerosols to the air. The salt and chemicals accumulated during the years when the Aral Sea received the salts, fertilizers, pesticides, herbicides, and other chemicals washed away from the irrigated massifs of the region, over the adjacent areas^28,30^. As a result, the most apparent positive trends over Central Asia have been reported near the desiccating Aral Sea^31,32^, which are likely due to increased dust emissions, as the feature is unlikely to be represented in most chemical transport models^33^. Overall, all these conditions have marked the aridification trend of Central Asia, thus creating a favorable environment for extreme DS formation.

Most alarmingly, on November 4, 2021, a very strong DS was developed in Central Asia and caused extreme deterioration of air quality in several countries^34^, seemingly affecting the health of millions of people. This DS has been widely discussed in the mass media of the Central Asian countries, while its air quality/synoptic effects demonstrated^34^. However, the chronology of the DS, its spatial coverage, and the magnitude of its effects; all remained unexplored due to the use of coarse-resolution remote sensing and limited ground-based observations. In this light, Central Asia is only scantily covered by ground-based observations of aerosols with huge observational gaps within. This prompts the use of high-resolution spaceborne remote sensing of aerosols, which can be combined with ground-based observations to leverage the examination of severe DS in the region. For instance, the Ultraviolet Aerosol Index (UVAI) was initially introduced in the late 1990s as a concept based on observations made by the Total Ozone Mapping Spectrometer (TOMS) sensors^2,35,36^. Since then, the UVAI has been extended to include measurements from the Ozone Monitoring Instrument (OMI) and was numerously used to detect absorbing aerosols in the atmosphere^37^ and to examine mineral dust^2,38–41^. As the UVAI is retrieved from the measured radiances, a priori assumptions about aerosol composition are not required for its calculation, thus refuting any undesirable uncertainties in the assumed aerosol optical properties^33,35–37,42^. We suggest that high-resolution spaceborne UVAI product from TROPOspheric Monitoring Instrument (TROPOMI) can be used to characterize previously unexplored features of strong DS in Central Asia and to estimate its spatial coverage.

On this basis, our study utilizes a unique combination of high-resolution TROPOMI UVAI with ground-based meteorological observations and Lagrangian particle modelling to explicitly characterize one of the strongest DS in the history of Central Asia, which occurred on November 4, 2021. For such characterization, we (a) use TROPOMI UVAI to track DS from its origin in the southern part of Kazakhstan and evaluate its spatial coverage (b) run the 48-hour back-trajectories by the Hybrid-Single Particle Lagrangian Integrated Trajectory (HYSPLIT) model to identify the dust pathways during November 4–14, (c) analyzed visibility at airports in the area of the storm’s influence to determine the time frame of the event, (e) used PM_10_ and PM_2*.*5_ measurements estimate the value of the dust concentration in the air, (f) analyzed the meteorological conditions in the area of the occurrence of the storm in the summer of 2021 to determine the DS drivers.

## Results

**Figure 1:**
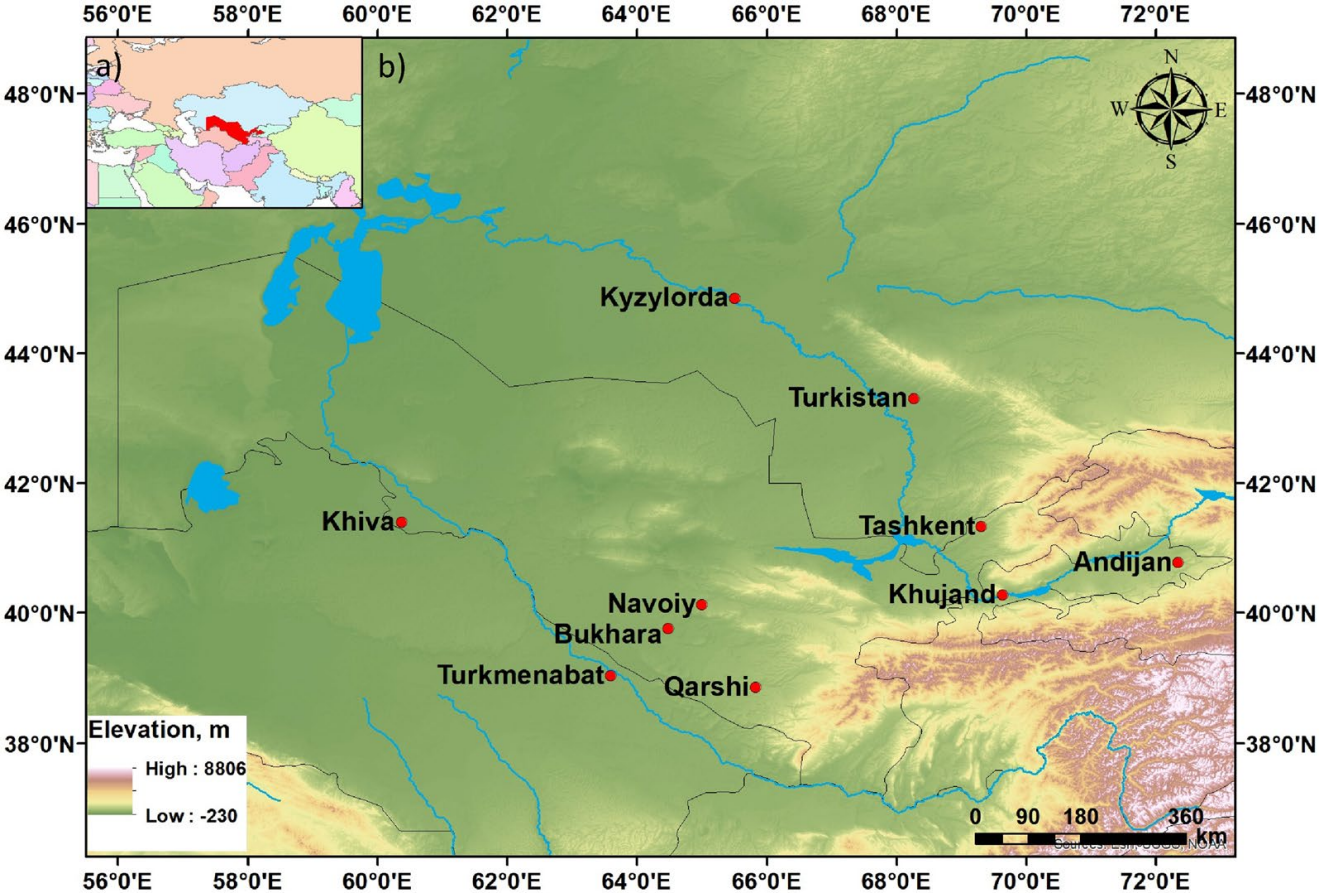
The study domain (**a**) Central Asia and (**b**) the Tashkent, Syrdarya, and Jizzakh regions, the Ferghana Valley regions of Uzbekistan, the Sughd region of Tajikistan, and the South Kazakhstan region of Kazakhstan. The map was created using ArcMap 10.8 obtained from website https://desktop.arcgis.com/.

The domain of the study is the central and eastern parts of Uzbekistan and adjacent regions of Kazakhstan and Turkmenistan, which are located in Central Asia (Fig. 1). The northern, northwestern, and western parts of the domain are flat. While the northeastern, eastern, and southern parts are occupied by the mountain systems of the Western Tien Shan and Hissaro-Alay. The plain part includes the steppe territories of Southern Kazakhstan, the Eastern Kyzylkum, the Mirzachul steppe, the Chirchik-Akhangaran Valley, and the Fergana Valley. The highest elevation is 5679 m altitude above sea level. The flat areas of the study region are characterized by dry, pronouncedly continental climates, while the mountainous areas have a temperate climate. In the summer months, the temperature maximum in the deserts can exceed + 45 °C, while the temperate minimum of winter months in the north can drop below − 36 °C. Depending on the physical and geographical conditions, the humidification regime exhibits strong variability. In the flat northern and northwestern areas, the annual precipitation is only 150–200 mm, while in the foothill areas, it can reach 200–600 mm and even 1000 m in the mountains. In spring, summer, and autumn, the northern, north-western, and western areas manifest as the hotspots of DS development. The average duration of DSs in the region exceeds 20 days. In some years, DSs can even last from 40 to 110 days^43^. The local soils consist of sand and light texture particles. The average size of sand particles ranges from 90–100 to 170–270 microns and can quickly be lifted into the air by the wind^44^. In the area of the neck of the Fergana Valley, the average duration of DS is 20 days, while in the lowland areas it is about 5–10 days^21^.

**Figure 2:**
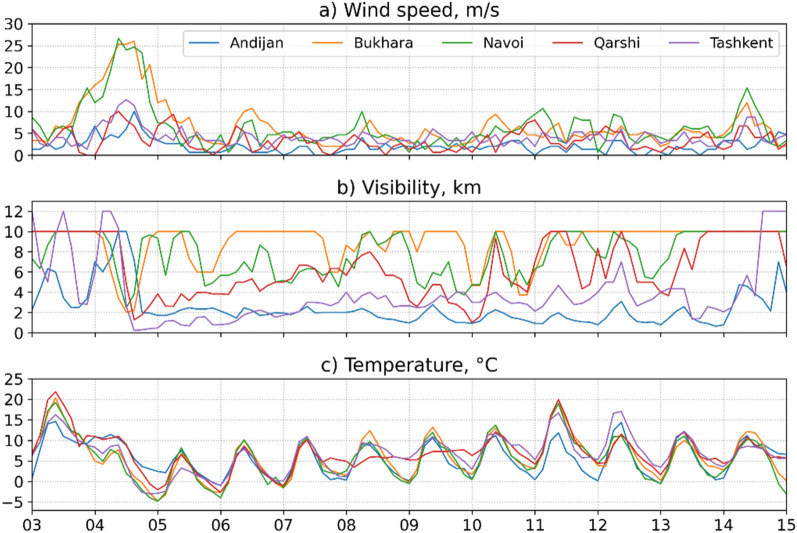
The 3-hourly averaged (**a**) wind speed, and (**b**) visibility range observed at the Andijan, Bukhara, Navoi, Qarshi, and Tashkent airports.

As mentioned in the introduction, it is essential to reconstruct the chronology of the strong DS of interest for the physical characterization of the event and its consequences. A previous study indicated that DS started in the afternoon of November 4, when the front part of the cold polar anticyclone front from the Ural–Volga regions invaded the territory of Uzbekistan through the southern regions of Kazakhstan^34^. Interestingly, we detected the prolonged strong winds in the study area, as shown in Fig. 2a (24h in Tashkent and Andijan; 36h in Bukhara and Navoi). The maximum average values reached 10–12 m/s in the cities of Tashkent and Andijan and up to 20 m/s in the cities of Bukhara and Navoi (all the cities are in Uzbekistan).

**Figure 3:**
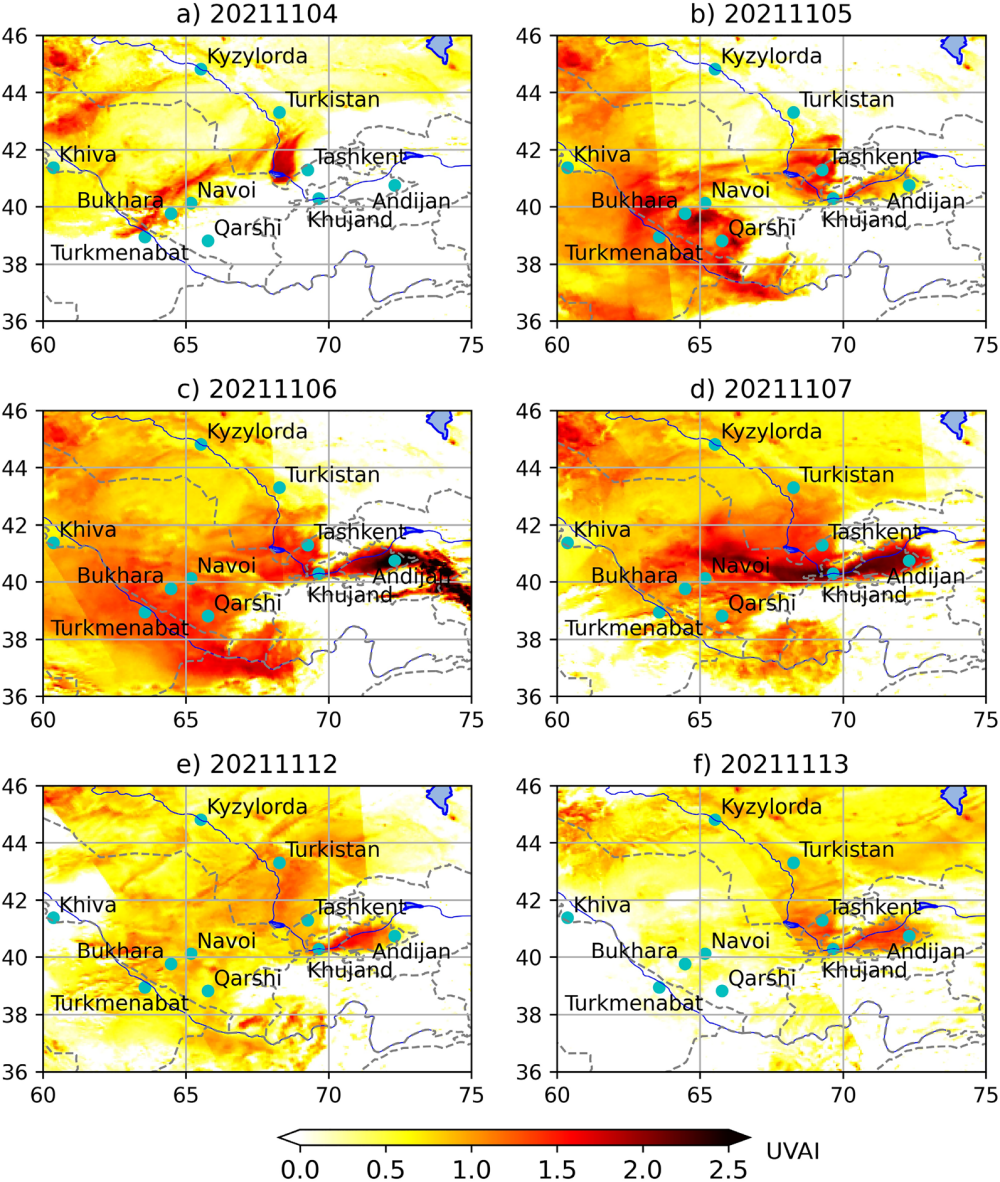
The DS development in the period 4–7 and 12–13 November 2021 based on the TROPOMI UVAI. The maps were created using a Python library for cartographic visualizations Cartopy (Version 0.21.1) obtained from website https://pypi.org/project/Cartopy/.

As a result, dust particles from the surface in areas with a desert landscape were lifted in the air, thereby forming a dust veil suspended into the air. Although the MODIS-acquired true-color image has been used to track the movement of dust flows in the initial phase (November 4) in the previous study on this DS^34^; the information about the following days is lacking because heavy clouds critically hampered any detailed analysis using MODIS. To elucidate this previously unexplored pattern, we used high-resolution estimates of UVAI from the TROPOMI sensor, benefiting from weaker sensitivity to clouds and superior cloud filtering, compared to earlier missions. As a result, high-resolution UVAI clearly unveiled two dust plumes moving from the Turkestan region (south Kazakhstan) in a south-westerly direction (Fig. 3a). Moreover, a more horizontally elongated western plume spreading over a flat area with a higher speed of movement (up to 25 m/s) was detected. As a result, the dust layer consequently covered the Bukhara-Turkmenabad-Qarshi region (Uzbekistan) and a large area further south from November 5–6. The large-scale regional dispersion further substantially reduced the dust concentration at individual points, thereby gradually improving the air visibility range after the passage of the dust front (Fig. 3d).

**Figure 4:**
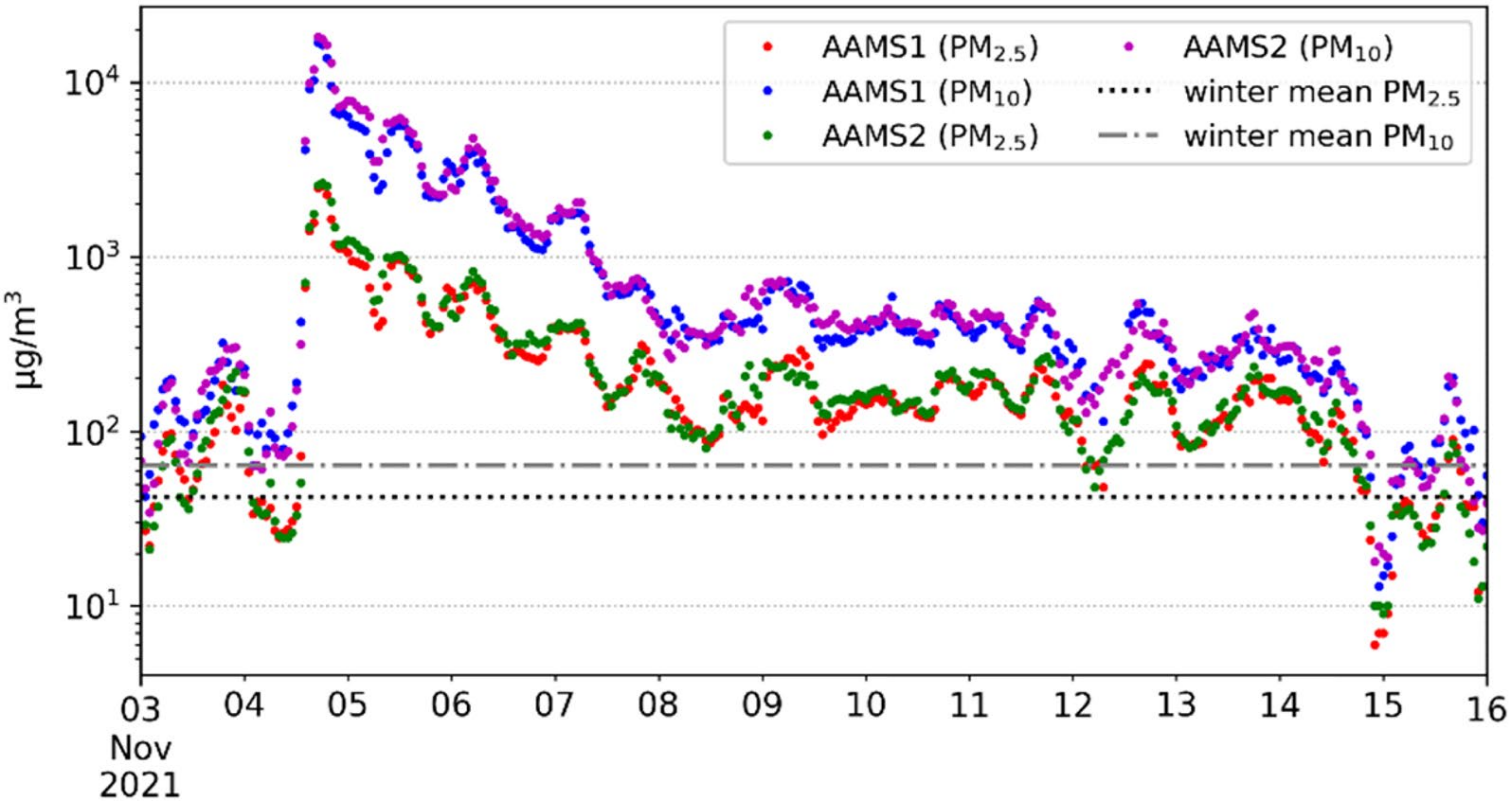
Observed PM_10_ and PM_2*.*5_ concentrations [µg/m^3^]. Symbols represent concentration at AAMS1 and AAMS2 observation posts located in the central part of Tashkent during November 3–16, 2021. Lines show mean values derived during winter periods of a long-term monitoring campaign undertaken at an urban background site of Tashkent city during December 1, 2011–November 30, 2014^45^.

Unlike the relatively simple pattern of the first plume we reported above, the second plume exhibited a more complex structure. More specifically, it deviated to the southeast and spread toward the Tashkent-Khujand-Andijan region. On the night of November 4–5, a dust veil covered the Tashkent region and entered the Ferghana Valley (both—Uzbekistan) through the narrow Khojent gorge in the Sughd region of neighboring Tajikistan. A salient increase of UVAI values indicated that the acute phase of DS in this area occurred from a period of November 5–7 (Fig. 3b–d) up to November 12–13 (Fig. 3e–f). Since DS most severely affected the region during the acute phase, we further elucidate this phase, its chronology, and effects. From a wind perspective, gusts reached 20 m/s at 18:00 Tashkent time (13:00 UTC). Then, circa 18:21 (13:21 UTC), a plume of suspended dust particles covered the city and the visibility range plummeted below 200 meters at Tashkent airport (Fig. 2). The ground-based aerosol observations from two locations in Tashkent (PM_2.5_ and PM_10_; the most common indicators of air quality) demonstrated that air quality has quickly deteriorated in Tashkent, driven by the effects of DS (Fig. 4) At the Automatic Air Monitoring Station (AAMS) #2, PM_10_ sharply rose from 312 µg/m^3^ (18:00) to 4 575 µg/m^3^ (19:00). Eventually reached the extreme values of 18 485 µg/m^3^ 21:30 (16:30 UTC). The PM_2*.*5_ variation followed a similar trend by rising from 51 µg/m^3^ at 18:00 to 705 µg/m^3^ at 19:00 and ultimately reaching the maximum of 2 712 µg/m^3^ at 22:18 (17:18 UTC). Strikingly, these maxima represent ~260 and 60-fold exceedances of the regional long-term average values of PM_10_ (63.7 µg/m^3^) and PM_2*.*5_ (42 µg/m^3^), respectively^45^. At the same time, we observed slightly lower aerosol concentrations at AMS1, seemingly driven by the local wind conditions.

Fig. 4 shows a decrease in the concentration of PM_10_ and PM_2*.*5_ during the next four days on November 5–8 under low wind conditions (2–3 m/s) of the north-northeast direction mainly. At the same time, large diurnal fluctuations were discerned, which were apparently caused by a locking inversion in the surface layer of the atmosphere. The intensity of self-purification of the atmosphere slows down in the following days on November 8–14, when concentrations exhibited lower variability. Finally, on November 14–15, a sharp decrease in PM_10_ and PM_2*.*5_ values was observed, which was caused by snow precipitation. The concentration value approached the state close to the average long-term winter values of 63.7 and 42 µg/m^3^ for PM_10_ and PM_2*.*5_, respectively^45^.

Note that the World Health Organization (WHO) states both coarse (PM_10_) and fine aerosol particles (PM_2*.*5_) should be classified as priority air pollutants^46^. These particles irritate the respiratory tract and can exacerbate or even trigger such diseases as bronchitis, emphysema, and cardiovascular dysfunctions. Notably, our reported averages significantly exceed the WHO air quality guidelines of 20 and 10 µg/m^3^ for the annual mean PM_10_ and PM_2*.*5_, respectively^22^. Unsurprisingly, such air quality deterioration caused by severe DS in Tashkent on 4 November 2021 inflicted immediate adverse health effects among the local population. Indicatively, we found that the ambulance service received 687 calls from inhabitants in Tashkent seeking help due to respiratory problems^34^.

**Figure 5:**
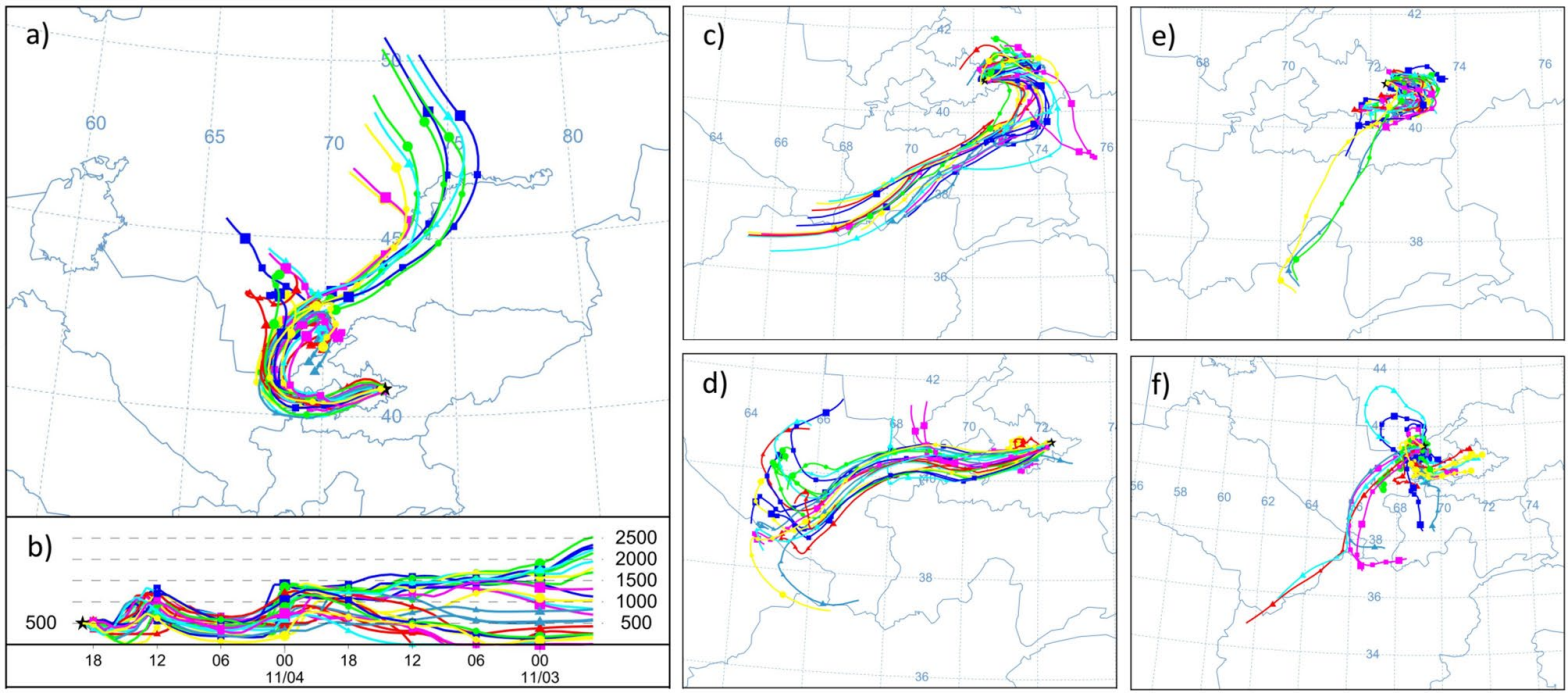
The HYSPLIT model backward trajectories are ending at Andijan on (**a**, **b**) November 4, (**c**) November 6, (**d**) November 14, (**e**) November 12, and (**f**) in Tashkent on November 12, 2021. For all panels, the time moment is 21:00 UTC, corresponding to 00:00 of the next day in local time.

We further analyzed the 48-hour trajectories of DS (Fig. 5a) using HYSPLIT. The path of the dust storm indicates that its origin was the southern part of Kazakhstan before it propagated to the Fergana Valley. Around the afternoon of November 4, the near-surface wind speed reached 10–20 m/s, thereby plaguing the visibility in Andijan and Tashkent. The visibility reduction events in these cities occurred with a temporal difference of ~ 4–5 h, while the physical distance between the cities is ~ 260 km. The sandstorm area of occurrence was accurately derived from the position of the yellow-red trajectories originating at the Earth’s surface (Fig. 5b). The next series of the trajectories, related to November 6 (Fig. 5c) revealed a dramatic change in the air circulation. This dramatic change trapped the polluted air containing aerosols in the area of the Fergana Valley. In the following days, the nearly calm atmospheric conditions favored the fine dust retention in the air (Fig. 5). This atmospheric pattern remained nearly the same until November 14 (Fig. 5d), when fresh air masses moving from the east provided sufficient ventilation.

**Figure 6:**
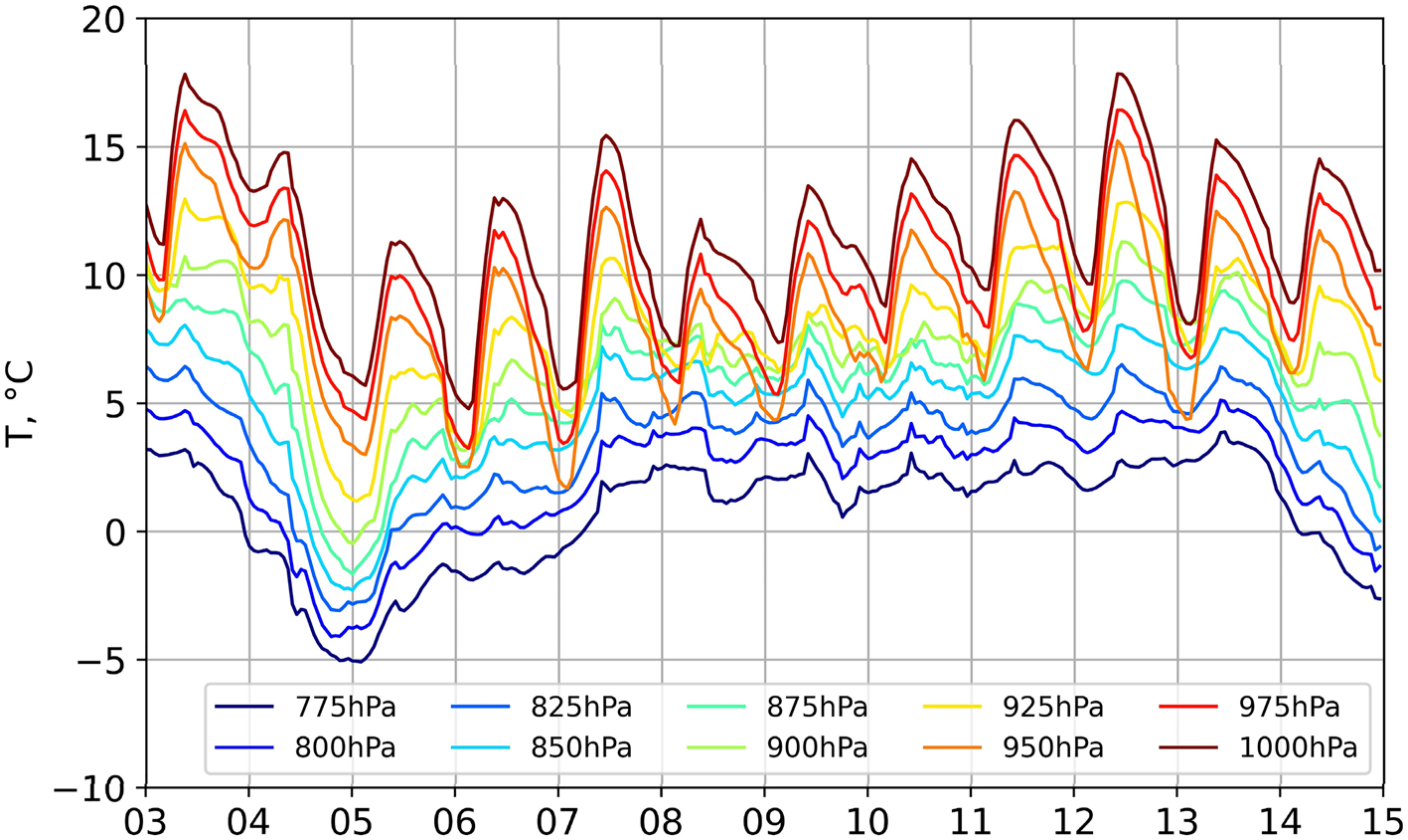
The hourly averaged air temperature at the levels of 1000–775 hPa derived from ERA5 reanalysis for Andijan in the period 3–15 November 2021.

Figure 5e, f shows the features of circulation over Andijan and Tashkent during the dust storm event on November 12. Over the Fergana valley surrounded by mountains, air ventilation is always very limited in the conditions of a weak wind of variable direction, which hampers and slows down the dust deposition from air during the event (Fig. 6e). Air quality in Tashkent is affected by two factors: (1) the inflow of dust-polluted air from the Ferghana Valley in the opposite direction relative to the beginning of the event on November 4 and (2) the transfer of relatively fresh air masses from the southern regions of Uzbekistan. Although the wind speeds were still low with the prevailing circulation of the polluted air, the visibility started to improve on November 8. Consequently, a significant difference in the visibility values for these cities was observed on November 12 (Fig. 2b). Note that the volumes of sand and dust that are being lifted into the air usually disperse and deposit on the ground somewhat quickly after the wind is weakening^47^. In this case, a mass of cold air forms an inversion layer, in which temperatures stop falling with the elevation. Thus, the temperature instead increases, thereby forming a meteorological condition favorable for smog formation (Fig. 6).

**Figure 7:**
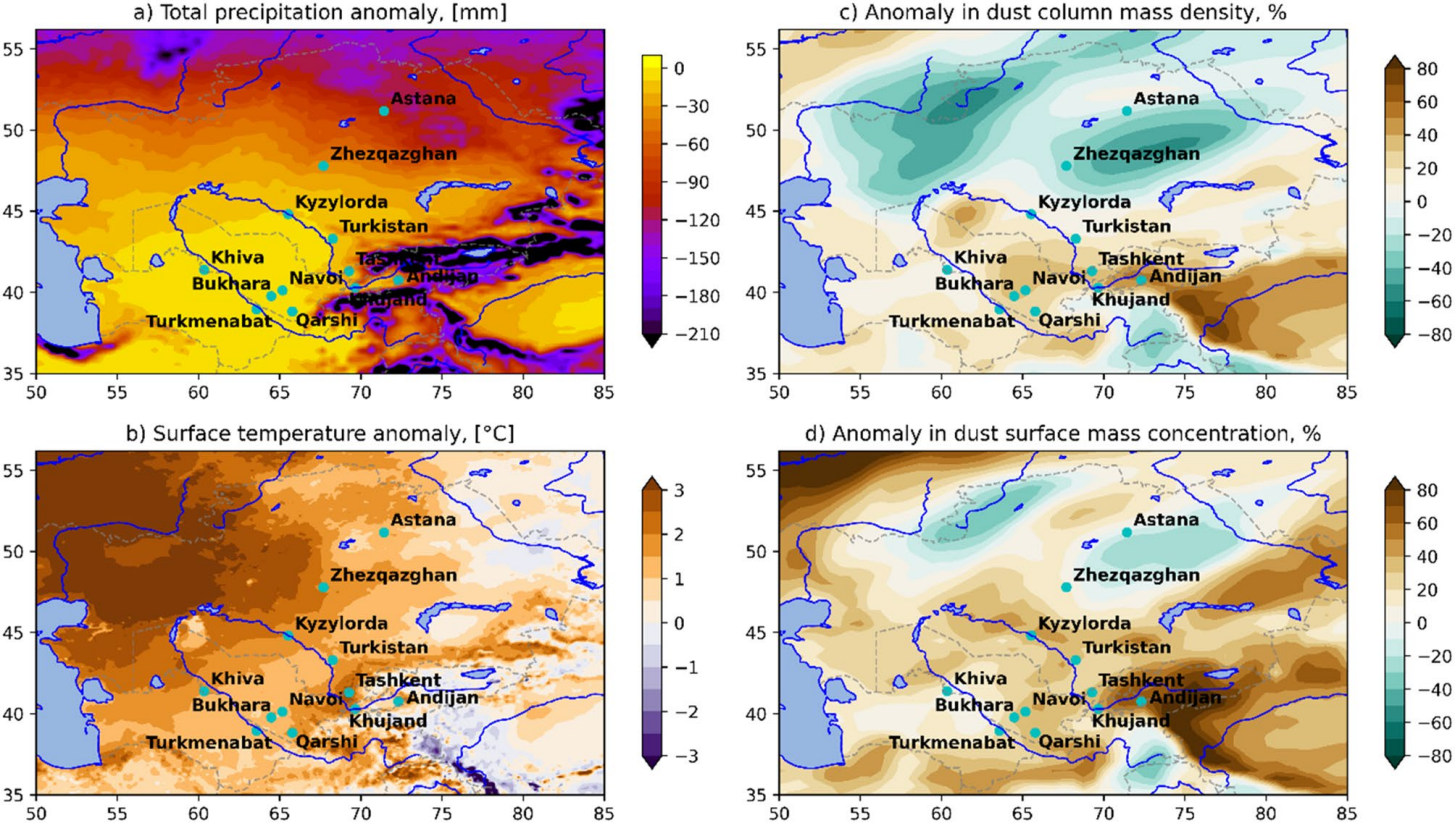
Deviation of (**a**) total precipitation (mm), and (**b**) the average surface temperature (°C) during the period June–August 2021 from the climatic norm for the period of 2000–2020 derived from ERA5-Land reanalysis. Anomaly (**c**) in dust column mass density (%), and (**d**) in dust surface mass concentration (%) in November 2021 relative to November 2000–2020 derived from MERRA-2 reanalysis. The maps were created using a Python library for cartographic visualizations Cartopy (Version 0.21.1) obtained from website https://pypi.org/project/Cartopy/.

Despite its vast size, Central Asia is very scarcely covered by ground-based meteorological observations. Therefore, the reanalysis, which is widely recognized as a valid proxy of observation^48^ can be used to alleviate these observational gaps in the studied region. For this purpose, we used the ERA5-Land, a reanalysis dataset providing a consistent view of the evolution of land variables at an enhanced resolution^49^. Specifically, we applied ERA5-Land to examine the meteorological conditions preceding the DS event over the domain extended to the northern part of Central Asia. Notably, nearly the entire area shown in Fig. 7 was affected by a strong drought in 2021^50^. In Kazakhstan, the heat wave that began in June 2021 in the Southern and Western regions of the country (Kyzylorda, Mangystau, and Turkestan regions) yielded record temperatures of 46.5 °C (recorded on 7 July 2021), with a base average of 28.3 °C^50^. In the suggested area of the DS occurrence, the average surface temperature in the period June-August 2021 was above the norm for the same period of 2000–2020 by 2–3 °C (Fig. 7b). While the total precipitation amount was much less (100 mm) than the norm (Table 1). The anomalously high air temperature in various regions of Kazakhstan the rapid runoff of rivers and reservoirs, while the soil dried down to a depth of 50 cm^50^. Thus, these processes caused the denudation of the soil cover because of the drying of vegetation and the intense loss of its moisture.

**Table 1:** The summer (June–August) mean temperature and precipitation for the period of 1981–2010 in comparison with the summer 2021 temperature and precipitation for selected stations in Kazakhstan.

Stations	Climatic mean temperature (in °C)	Temperature in 2021 (in °C)	Climatic mean precipitation (mm)	Precipitation in 2021 (mm)
Arys	25.2	27.3	49.6	10.7
Aul Turara	21.8	24.0	129.6	29.7
Bugun	25.0	27.7	69.9	4.6
Kazygurt	22.7	25.5	98.7	28.0
Karatau	22.6	24.3	68.4	21.0
Krasnayarskaya	19.3	24.1	125.5	–
Taraz	21.5	23.7	113.8	12.0
Tasaryk	19.9	21.1	167.7	52.0
Shuyldak	14.6	16.3	233.5	62.0
Shymkent	23.5	25.6	114.4	36.0

The MERRA-2 reanalysis^51,52^ shows a significant increase in the dust column mass density and in dust surface mass concentration during the summer-autumn of 2021 relative to the climatic value for the same period of 2000–2020. The highest values (40–60% more than climatic ones) are formed in the southern part of Kazakhstan adjacent to the border with Uzbekistan (values for November are shown in Fig. 7c). Moreover, the deviation of surface values is larger than the column one and reaches 60-80%, while in the Ferghana Valley more than 80% (Fig. 7d). A joint comparison of temperature and precipitation anomalies (ERA5-Land) and dust distribution (MERRA-2) indicates a significant relationship between summer heat waves and the intensity of dust storms in the southern part of Kazakhstan. A more detailed study of this is required, but it is beyond the scope of this article.

## Conclusion

Our study characterized one of the strongest dust storms in the history of Central Asia, which occurred on November 4, 2021, using a set of high-resolution TROPOMI UVAI spaceborne observations, ground-based aerosol measurements, and Lagrangian particle modelling. Most notably, the severe dust storm driven by the front part of the cold polar anticyclone front from the Ural–Volga regions hit the central and eastern parts of Uzbekistan on the evening of November 4, 2021, growing under favorable weather conditions into the worst since the country started keeping meteorological records in 1871. For the first time, we identified two plumes, which had spread out and a thick haze blanketed the central and eastern parts of Uzbekistan. Tashkent (the capital of Uzbekistan) and the Fergana Valley region of Uzbekistan were found to be the most affected by the severe dust storm. The ground-based observations at airports in the storm’s influence indicated that the visibility range plummeted down to 200 m as a very thick atmospheric dust layer was developed. In Tashkent, PM_10_ concentrations rose to 18,000 µg/m^3^, thereby exhibiting 260-fold exceedances, compared to the local long-term average value. The PM_2*.*5_ concentrations above 300 µg/m^3^ remained in the air for nearly 10 days, clearly demonstrating that the reported dust storm stood out with the extreme duration. During this period in the Andijan airports (Uzbekistan), the visibility range was below 2 km with frequent reduction down to 500 m, while in other cities hit by the dust storm, the visibility returned to the normal level within a few days. The 48-h HYSPLIT back-trajectories indicated a very rapid movement of air masses bringing dust from southern Kazakhstan at the initial moment of the event. In the following days, the trajectories exposed only a slight mixing of the dust-polluted air between Tashkent and Andijan under conditions of the shallow wind speed of variable direction, which lasted until November 14. The drivers of this extreme dust storm were elucidated for the first time. We found that a few months before the storm in the summer of 2021, an extremely strong heatwaves, characterized by unprecedented high temperatures (up to 46.5 °C) hit southern Kazakhstan; the origin area of the dust storm. The ERA5-Land data analysis revealed that the average air and surface temperature from June–August 2021 was 2–3 °C higher than the norm for the same period from 2000 to 2020. In contrast, the total amount of precipitation was only ~ 100 mm of rain, which is much lower than the norm. This long-lasting drought caused rapid runoff of rivers and reservoirs, and the soil dried up to a depth of 50 cm, resulting in the denudation of the soil cover due to vegetation drying out and losing its moisture. A joint comparison of temperature and precipitation anomalies from ERA5-Land and dust distribution MERRA-2 indicates a significant relationship between summer heat waves and the intensity of dust storms in the southern part of Kazakhstan.

The reconstructed chronology of the Central Asian dust storm from 2021 with the determined drivers and demonstrated air quality effects, immediately propagating to health effects among the local population indicates alarming evidence for the region. To be specific, this evidence demonstrates that extreme heatwaves and favorable meteorological conditions are the only two missing components for such extreme dust storms to become a regular event in Central Asia. Under the continuing global warming, such extreme dust storms can become recurring, thereby forcing this arid region, composed mostly of developing economies to suffer from excessive premature mortality and so-called eco-immigration. Nowadays most vulnerable populations of developing and emerging economies carry the burden of global warming effects (from crop degradation, and water scarcity to air quality-related mortality), and the potential exposure of Central Asia to extreme dust storms is concerning. To prevent such effects, not only the governments of Central Asian countries (given the institutional and economic challenges during the first decades of independence) should take actions to track and mitigate the effects of such storms. We suggest that international organizations which identify and mitigate climate change aftershocks should take early note of the emergence of another hotspot of environmental to minimize these risks. This can be done by directly and indirectly improving the atmospheric observational capabilities of Central Asian countries from the resource, manpower, and institutional viewpoints.

## Methods

### TROPOMI UVAI

The TROPOMI instrument on the Sentinel-5 Precursor (S5P) satellite launched on 13 October 2017 is a high-spectral-resolution spectrometer covering eight spectral windows from the ultraviolet (UV) to the shortwave infrared (SWIR) regions of the electromagnetic spectrum. The instrument operates in a push-broom configuration, with a swath width of about 2600 km on the Earth’s surface. The typical pixel size (near the nadir) is 5.5 × 3.5 km^2^ for all spectral bands, with the exception of the UV1 (5.5 × 28 km^2^) and SWIR (5.5 × 7 km^2^) bands^53,54^. TROPOMI provides the UVAI product that characterizes the content of ultraviolet-absorbing aerosols, such as dust and smoke, through UV channel inversion parameters^55,56^. The relatively simple calculation is based on wavelength-dependent changes in Rayleigh scattering in the UV spectral range where ozone absorption is very small. UVAI can also be calculated in the presence of clouds so that daily, global coverage is possible. To obtain the daily horizontal distribution of dust events, we utilized UVAI data. Since S5P/TROPOMI was unable to cover all of Central Asia in a single observation, we seamed together UVAI images of the entire day by selecting the maximum value of image elements at the image overlap. The data were acquired from the Sentinel-5P official website [https://s5phub.copernicus.eu/dhus/#/home (accessed on 18 April 2023)]. We only used credible data with data quality values greater than 0.8, as recommended by the satellite product’s official guidelines.

### The HYSPLIT model

The HYSPLIT model is a computer model developed by the US National Oceanic and Atmospheric Administration’s (NOAA) Air Resources Laboratory (ARL) used to compute the trajectories of air parcels, which helps in determining the direction and distance that air pollutants will travel^57,58^. Along with that, HYSPLIT is capable of computing air pollutant dispersion, chemical transformation, and deposition. One popular use of HYSPLIT is to establish whether high levels of air pollution at one location are caused by the transport of air contaminants from another location. HYSPLIT’s back trajectories, combined with satellite images, can provide insight into whether high air pollution levels are caused by local air pollution sources or whether an air pollution problem was blown in on the wind^34,59–61^. In this study, HYSPLIT air-mass back-trajectories were used at receptor sites in Central Asia: Tashkent (41.3° N, 69.26° E) and Andijan (40.74° N, 72.31° E) during the dust storm (4–14 November 2021). The model was driven by the archived three-dimensional National Centers for Environmental Prediction (NCEP) Global Forecast System (GFS) latitude-longitude grid data with the resolution of 0.25° through the web-based Real-time Environmental Applications and Display sYstem [READY; https://www.ready.noaa.gov/HYSPLIT_traj.php (accessed on 18 April 2023)]^62^. The trajectories were computed for a height of 500m AGL, the label interval was set to be six hours to track the path of the trajectory.

### Ground based observations

Regular observations of meteorological parameters (wind speed and direction, temperature, visibility range, etc.) are conducted at airports in Uzbekistan. For visibility range measurements the Islam Karimov Tashkent International Airport in Tashkent uses Vaisala’s MITRAS transmissometer, while the Andijan, Bukhara, Navoi, and Qarshi airports employ the Vaisala FD12 nephelometers. In Tashkent PM_10_ (the atmospheric Particulate Matter with particles smaller than 10 microns) and fine PM_2*.*5_ (particles smaller than 2.5 microns) are measured by optical dust analyzers Horiba APDA-372 installed at two observation posts located in the central part of Tashkent (AAMS1 and AAMS2). The APDA-372 dust monitor is specifically designed for indoor and outdoor air quality measurements and provides continuous and simultaneous measurements of the particle size distribution within the particle size range of 0.18–18 µm. We analyzed wind speed, temperature, and horizontal visibility range using meteorological monitoring data from Andijan, Bukhara, Navoi, Qarshi, and Tashkent airports to determine the moment of the DS passage and assess its intensity. The PM_10_ and PM_2*.*5_ observations are more reliable characteristics of air quality in the DS conditions.

### ERA5

We use the European Center for Medium Range Forecasts ERA5^63^ reanalysis at 0.25° × 0.25° horizontal resolution and 37 pressure levels vertical resolution from (1000 to 1hPa) with hourly sampling [https://cds.climate.copernicus.eu/ (accessed on 18 April 2023)].

### ERA5-Land

ERA5-Land has been produced by replaying the land component of the ECMWF ERA5 climate reanalysis. Reanalysis combines model data with observations from across the world into a globally complete and consistent dataset. ERA5-Land provides a consistent view of the water and energy cycles at the surface level from 1950 onwards, with a temporal resolution of 1 h. The native spatial resolution of the ERA5-Land reanalysis dataset is 9 km on a reduced Gaussian grid, which has been regridded to a regular latitude-longitude grid of 0.1° × 0.1°. Monthly-mean averages have been calculated to facilitate many applications requiring easy and fast access to the data when sub-monthly fields are not required [https://cds.climate.copernicus.eu/ (accessed on 18 April 2023)].

### MERRA2

MERRA-2 is the latest version of global atmospheric reanalysis for the satellite era produced by NASA Global Modeling and Assimilation Office (GMAO) using the Goddard Earth Observing System Model (GEOS) version 5.12.4. The dataset covers the period of 1980-present with the latency of ~3 weeks after the end of a month at 0.5° × 0.625° horizontal resolution. In this work we use a time-averaged 2-dimensional monthly means data collection of dust column mass density (DUCMASS) and dust surface mass concentration (DUSMASS) downloaded from [https://search.earthdata.nasa.gov/ (accessed on 30 May 2023)].

## Data Availability

The sources of the used data are mentioned in the “Methods”. Other generated data and tools are available upon request by any user; they are both freely available to any researcher wishing to utilize them for non-commercial purposes, without breaching participant confidentiality. All data processing codes were developed using Python and also can be made available upon request to the corresponding authors. To request the data from this study, please contact N.B.E. (bnishonov@mail.ru) or D.B.A. (d.belikov@chiba-u.jp).
